# Combined Distal Femoral Osteotomy and Medial Patellofemoral Ligament Reconstruction for Patellar Instability and Genu Valgus: A Case Report and Literature Review

**DOI:** 10.1111/os.70057

**Published:** 2025-05-31

**Authors:** Bin Zhao, Zijian Lian, Xuan Jiang, Songqing Ye, Haohao Bai, Wei Luo, Xinlong Ma

**Affiliations:** ^1^ Tianjin Hospital Tianjin University Tianjin China

**Keywords:** derotational distal femoral osteotomy, genu valgus, lateral opening wedge distal femoral osteotomy, medial patellofemoral ligament, patellar instability

## Abstract

**Background:**

Medial patellofemoral ligament (MPFL) reconstruction alone is not effective for patellar instability associated with anatomic abnormalities of lower limbs. In this article, we report a case of complex lower limb malformations, including genu valgus, lower limb shortening, and increased femoral anteversion angle. In addition to MPFL reconstruction, we performed a rare osteotomy named combined distal femoral osteotomy (CDFO), which combined the characteristics of lateral opening wedge distal femoral osteotomy (LOWDFO) and derotational distal femoral osteotomy (DDFO).

**Case Presentation:**

We report the case of a 52‐year‐old female with left knee pain, valgus, and instability who was diagnosed with patellar instability and valgus knee osteoarthritis. Considering the patient's relatively young age, a hip‐knee‐ankle angle (HKA) of 194°, a mechanical lateral distal femoral angle (mLDFA) of 77.5°, a shortened left lower limb of 7 mm, an increased femoral anteversion angle (FAA) of 37.4°, and a patellar instability, we performed MPFL reconstruction and CDFO treatment. In this procedure, computer‐aided design (CAD) combined 3D‐printed osteotomy guide‐assisted CDFO and MPFL reconstruction were performed. At 6‐month follow‐up, the patient achieved satisfactory results, with an HKA of 180°, an mLDFA of 90°, an FAA of 15°, the same length of lower limbs, and patellar stability. There was significant improvement in her left knee pain, function, and patellar stability.

**Conclusions:**

To our knowledge, this rare pattern of patellar instability has not been previously described. Careful analysis of anatomic abnormalities is of great clinical significance and can better guide clinical treatment. CDFO may be an acceptable treatment for patellar instability with genu valgus and increased femoral anteversion angle.

## Graphical abstract

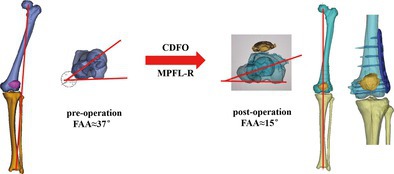

## 1 Background

Patellar instability can be caused by various factors, including soft tissue factors and bone factors [1]. Among them, soft tissue factors mainly include medial patellofemoral ligament (MPFL) injury [2]. Bony factors include genu valgus deformity, tibial tubercle deviation (tibial tuberecle‐trochleo groove, increased TT‐TG value), and femoral trochlea deformity, as well as femoral and tibial rotation deformity [3]. Accurate and comprehensive evaluation of the causes of patellar instability is the basis for formulating a reasonable surgical plan. Studies have shown that the selection of surgical methods for patellar instability should fully consider the bone pathogenic factors, and simple MPFL reconstruction often leads to treatment failure [4, 5].

Lateral opening wedge distal femoral osteotomy (LOWDFO) is one of the feasible surgical methods for patellar instability complicated with genu valgus. LOWDFO improves patellar trajectory by correcting the tibial tubercle and reducing the *Q* angle [6]. Rotational deformity of the lower limbs is also one of the causes of patellar instability and is one of the factors that are easily overlooked [7]. This deformity includes the anteversion of the femur and the external rotation of the tibia. It has been suggested that derotational distal femoral osteotomy (DDFO) should be performed in conjunction with MPFL reconstruction when the femoral pronation deformity is > 25° [8]. However, the cases with both valgus deformity and rotational deformity have not been reported.

In this article, we report a case of complex lower limb malformations, including genu valgus, lower limb shortening, and increased femoral anteversion angle. In addition to MPFL reconstruction, we used the integrating 3D‐printed osteotomy guide to perform a rare osteotomy named combined distal femoral osteotomy (CDFO), which combined the characteristics of LOWDFO and DDFO. To our knowledge, this is the first time that this rare pattern osteotomy based on integrating 3D‐printed osteotomy guide has been reported, and there are no other similar cases in the English literature.

## 2 Case Presentation

The patient is a 52‐year‐old female with a weight of 50 kg, height of 160 cm, and body mass index (BMI) of 19.5 kg/m^2^. She complained of left knee joint swelling and pain with limited mobility for 40 years, aggravated for 2 years. Left knee patellar dislocation occurred during sports exercise 40 years before admission, which was self‐reduced. One year after conservative treatment, the patient complained of increasing pain in the left knee and was unable to walk more than 500 m every day. Then, she was transferred to our department.

### 2.1 Physical Examination

**FIGURE 1:**
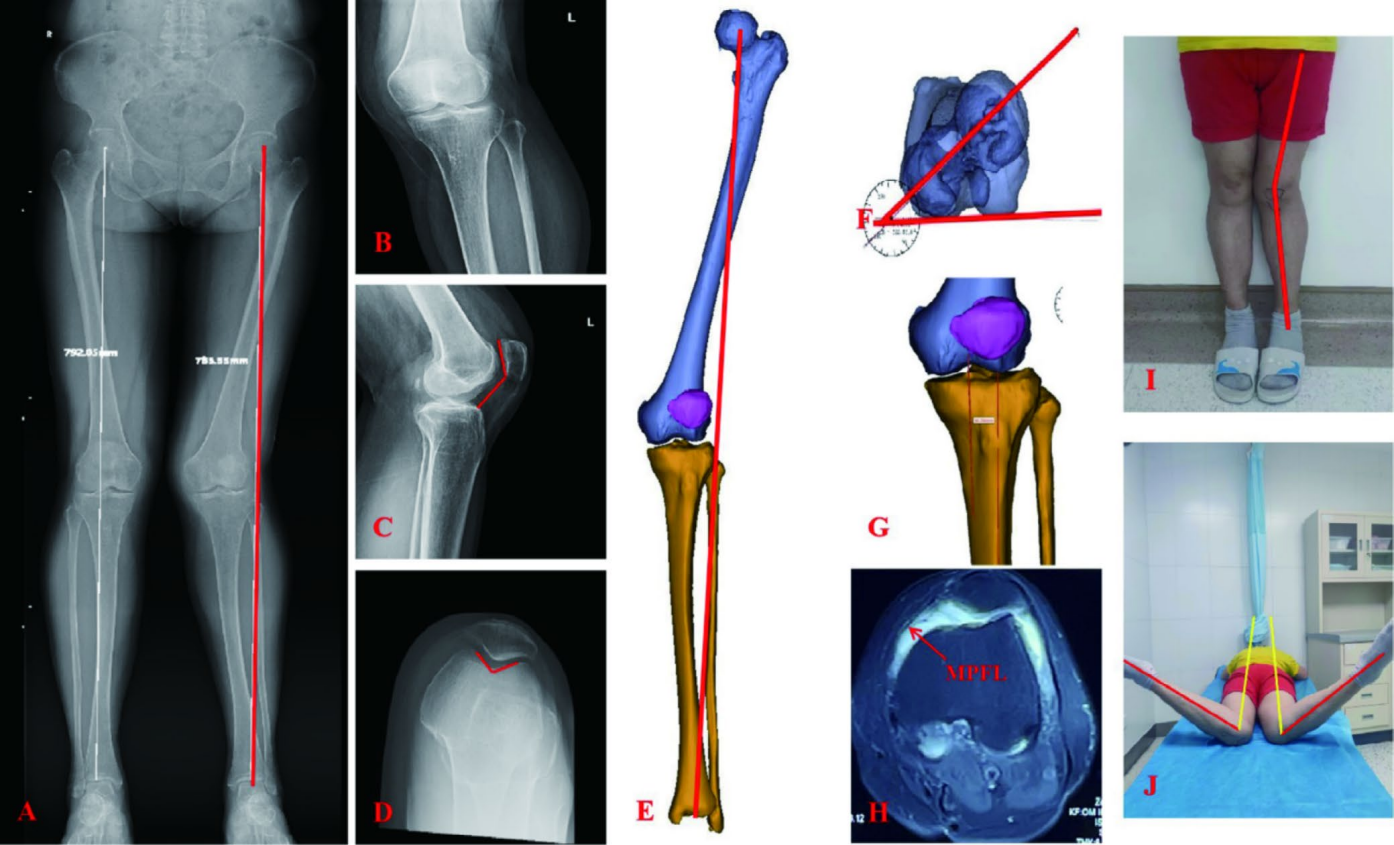
Preoperative photographs and fluoroscopy. (A) The preoperative standing X‐ray of the lower limbs. (B) The preoperative X‐ray of the knee in anteroposterior position. (C) The preoperative X‐ray of the knee in lateral position. (D) The preoperative X‐ray of the knee in 90° axial position. (E) The preoperative 3D‐CT revealed genu valgus of the left lower limb. (F) The preoperative 3D‐CT showed that the femoral anteversion angle was 37.4°. (G) The preoperative 3D‐CT showed that the tibial tuberosity‐trochlear groove distance was 16.8 mm. (H) The preoperative MRI revealed the injury of the MPFL. (I) Preoperative appearance revealed genu valgus of the left lower limb. (J) Preoperative appearance suggested that the left hip internal rotation angle was 70°.

Left knee genu valgus; Range of Motion (ROM): 0°–130°; lateral femoral condyle tenderness (+); lateral knee space tenderness (+); patellar extrapolate fear test (+); J sign (+); McMurray sign (+); anterior drawer test (−); Lachman test (−); 0° and 30° lateral stress test (−). The Western Ontario and McMaster Universities Arthritis (WOMAC) Index was 37. See Figure 1 for details.

### 2.2 Radiological Examination

Magnetic resonance imaging (MRI) of the right knee showed abnormal signal of the MPFL, abnormal signal of the left lateral femoral condyle, lateral tibial plateau, and local subarticular surface of the patella, and joint effusion (Figure 1H). Computed tomography (CT) showed poor alignment of the patellofemoral joint (Figure 1E). Full‐length DR film of both lower limbs showed left knee genu valgus (Figure 1A).

The patient underwent CT of the lower limbs in a low‐dose set (Lightspeed VCT; GE, Las Vegas, NV), and the layer thickness of the CT scan was 0.625 mm. The DICOM‐formatted CT images were imported into Mimics 21.0 software, where the bone was extracted by different thresholds, and the region‐growing function was applied to segment the femur, tibia‐fibula, and patella and generate the corresponding 3D skeletal models. All parameters were measured on the 3D skeletal models. The hip‐knee‐ankle angle (HKA) was 194°. The mechanical lateral distal femoral angle (mLDFA) was 77.5°. The mechanical medial proximal tibial angle (mMPTA) was 88.8°. The joint line convergence angle (JLCA) was 1°. The tibial tuberosity‐trochlear groove distance (TT‐TG) was 16.8 mm. The Caton‐Deschamps index was 1.12. The femoral anteversion angle (FAA) was 37.4°. The shortened length of the left lower limb was 7 mm.

### 2.3 Final Diagnosis

Left knee genu valgus and torsion deformity with lateral interventricular osteoarthritis and patellar instability. It was comprehensively considered that the main cause of patellar instability was the medial soft tissue injury of the knee joint and the valgus deformity and rotation deformity of the femur. Because the patient also had left lower limb shortening, the decision was made to perform MPFL reconstruction combined with CDFO, which combined the characteristics of LOWDFO and DDFO.

## 3 Operative Procedure

**FIGURE 2:**
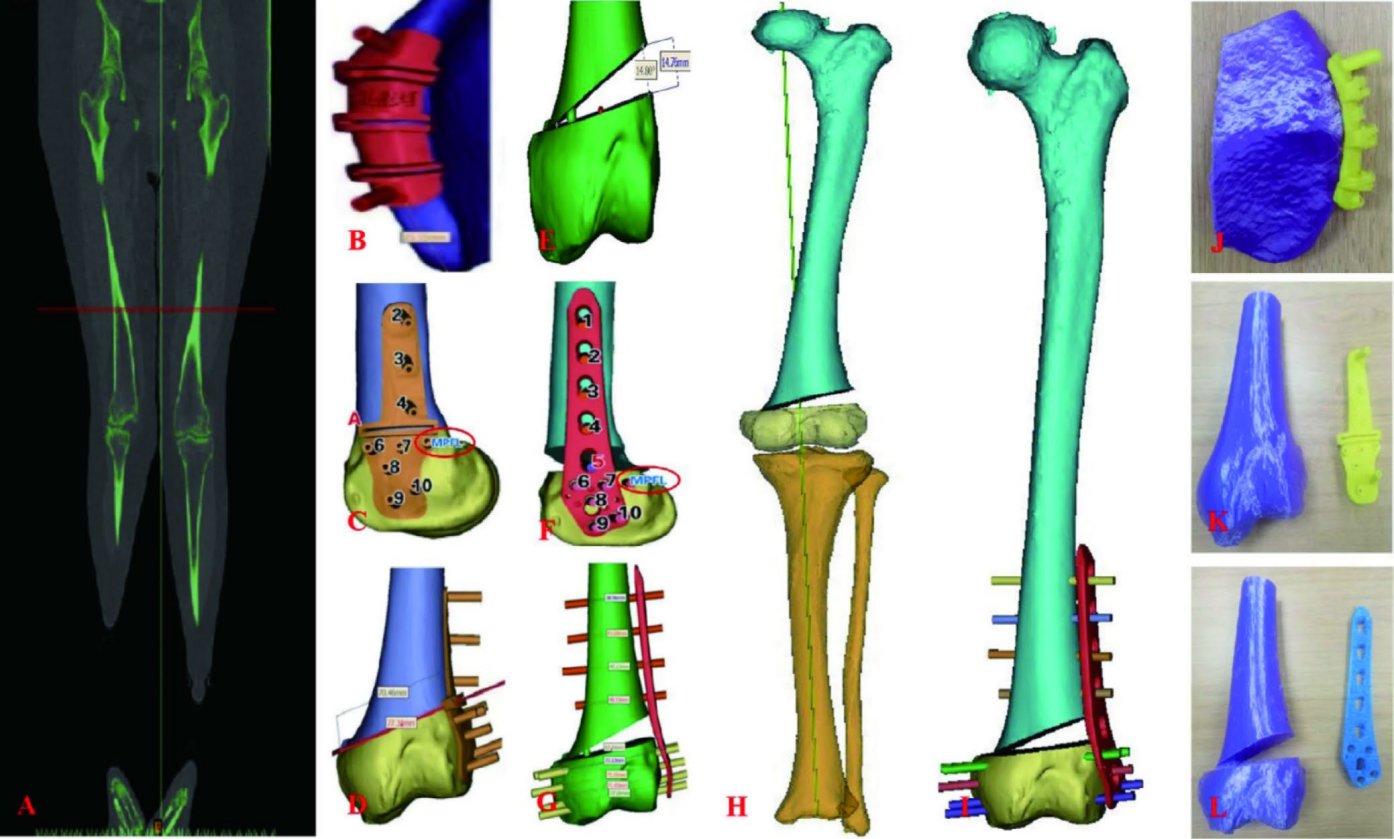
The 3D model reconstruction and simulated surgery. (A) CT images of the lower limbs. (B) Design of iliac crest osteotomy guide. (C) Design of femoral crest osteotomy guide. On the guide plate, we designed the symmetry point of MPFL femoral stop. The opposite side of this point is the femoral stop for MPFL reconstruction. (D) Determination of the osteotomy plane. (E) The end of the osteotomy is stretched 14.8°, that is, 14.76 mm. (F, G) Design of plate and screws. (H) Simulated surgery showed that the lower limb alignment returned to normal. (I) Simulated surgery indicated that the internal fixation position was satisfactory and the plate was attached well. (J–L) 3D printing guides, plates, and limb models.

The operation was performed using a computer‐aided design (CAD) combined 3D‐printed osteotomy guide based on the authors' previous studies [9, 10, 11]. The simulated surgery was performed on a 3D model in Mimics 21.0 (Figure 2).

General epidural anesthesia was administered according to individual patient conditions. All patients received intravenous cefazolin sodium (1.0 g) 30 min before surgery. An inflatable lower‐limb tourniquet was placed around the root of the thigh and the pressure adjusted to 45 kPa. A diagnostic knee examination was performed before the operation. Arthroscopic examination showed that the medial patellar cartilage injury was observed and cleaned with a shaver. The medial compartment cartilage was intact. Lateral compartment cartilage was worn and degenerated. According to the Outerbridge classification of articular cartilage injury under arthroscopy, the lateral compartment cartilage was grade III–grade IV. The femoral trochlear groove was shallowly developed, and the patella was obturated to the lateral femoral condyle. Abnormal patellar tracking was observed during knee flexion.

**FIGURE 3:**
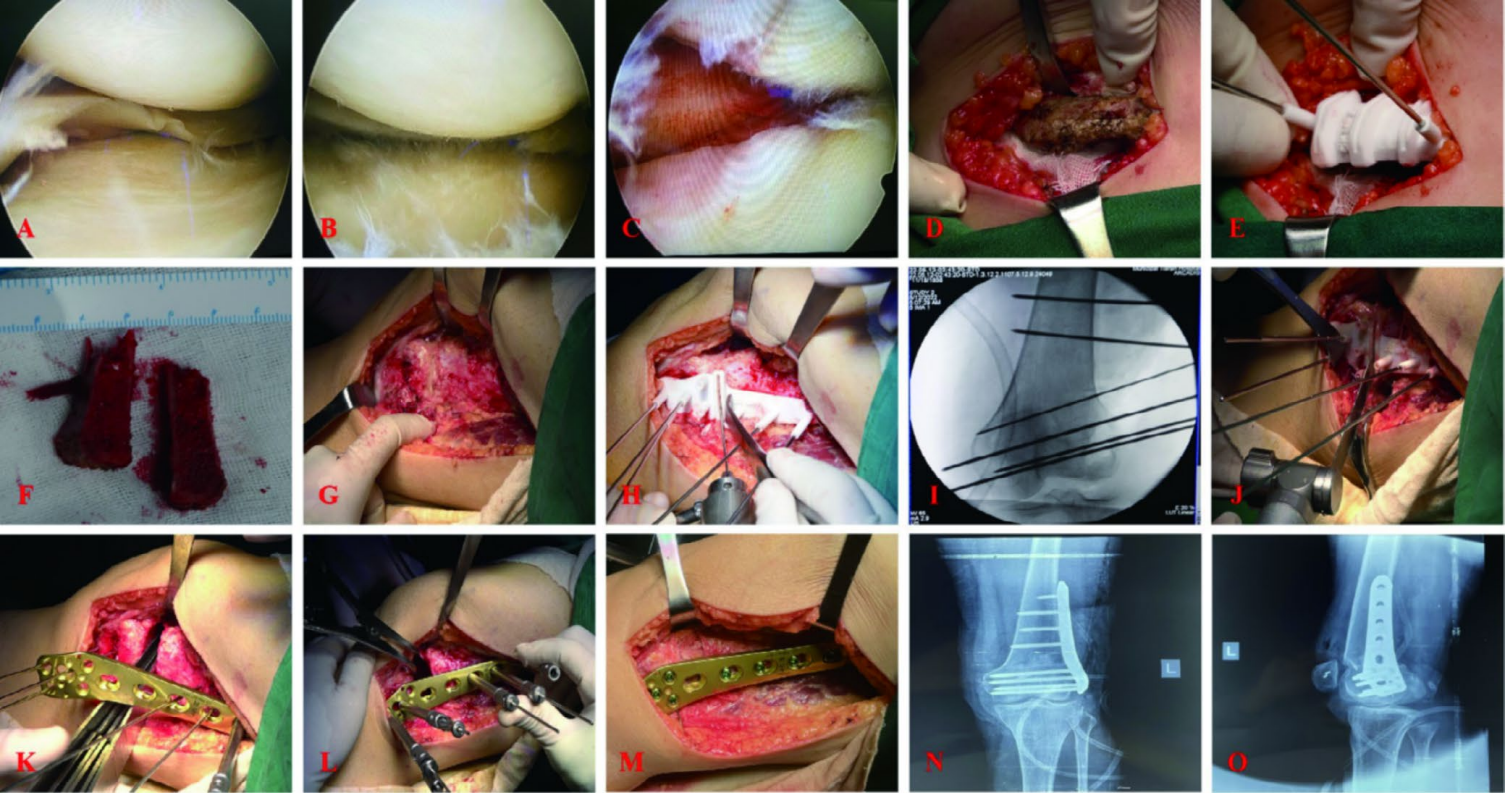
Intraoperative photographs and fluoroscopy. (A) Lateral compartment under arthroscopy. (B) Medial compartment under arthroscopy. (C) Patellofemoral joint under arthroscopy. (D) Expose ilium. (E) The iliac crest osteotomy guide was placed. (F) Two trapezoidal iliac bone fragments were removed completely. (G) Expose the distal femur. (H) The femoral osteotomy guide was placed. (I) The position of the femoral osteotomy guide was observed under fluoroscopy. (J) Distal femoral osteotomy was performed with a swinging saw. (K) A bone chisel was used to open the distal femur and a plate was placed. (L) The distal femur was derotated and laterally opened with a distractor. (M)The plate was fixed and the iliac bone was implanted into the osteotomy. (N) Intraoperative X‐ray of the knee in anteroposterior position. (O) Intraoperative X‐ray of the knee in lateral position.

To promote the growth of the osteotomy end, we decided to harvest autogenous iliac crest and implant it into the osteotomy end. According to the preoperative planning, the iliac bone removal surgery was performed with the assistance of a 3D printing guide plate. The two trapezoidal iliac fragments could be completely embedded in the osteotomy site. CDFO was performed using a three‐dimensional printed guide based on the authors' previous studies [9, 10, 11]. A locking plate (TomoFix, Ruihe Medical, Shanghai, China) was used. MPFL reconstruction using autologous ipsilateral semitendinosus tendon or gracilis tendon was performed after the osteotomy was completed. On the guide plate, we set the position of the femoral stop during MPFL reconstruction. An additional Kirkner pinhole is prearranged on the guide plate, and the opposite side of this hole is the femoral stop during MPFL reconstruction. See Figure 3 for details.

Tranexamic acid (TXA) 1 g was injected intravenously 10 min before the release of the tourniquet. The wound was then rinsed and hemostasized after the release of the tourniquet. After the incision was closed, 1 g TXA was injected into the osteotomy site. Finally, elastic bandages were applied to compress the wound.

## 4 Postoperative Management and Follow‐Up

Anticoagulation therapy and infection prevention were routinely administered. Enoxaparin sodium (4000 AxaIU; Sanofi‐aventis, Paris, France) was injected subcutaneously 12 h postoperatively and was continued for 4 days. Cefazolin sodium (1 g) was administered intravenously twice within 24 h of the operation. After recovery from anesthesia, the patients were instructed to perform ankle pump and straight leg elevation exercises. On postoperative day 2, the knee joint of the affected limb was allowed to flex and extend, and standing on the affected limb was permitted with bedside assistance without weight bearing. The patient can carry partial weight with crutches on postoperative day 3, gradually increase the weight from 4 weeks after surgery, and walk without crutches bearing weight at 8 weeks after surgery.

**FIGURE 4:**
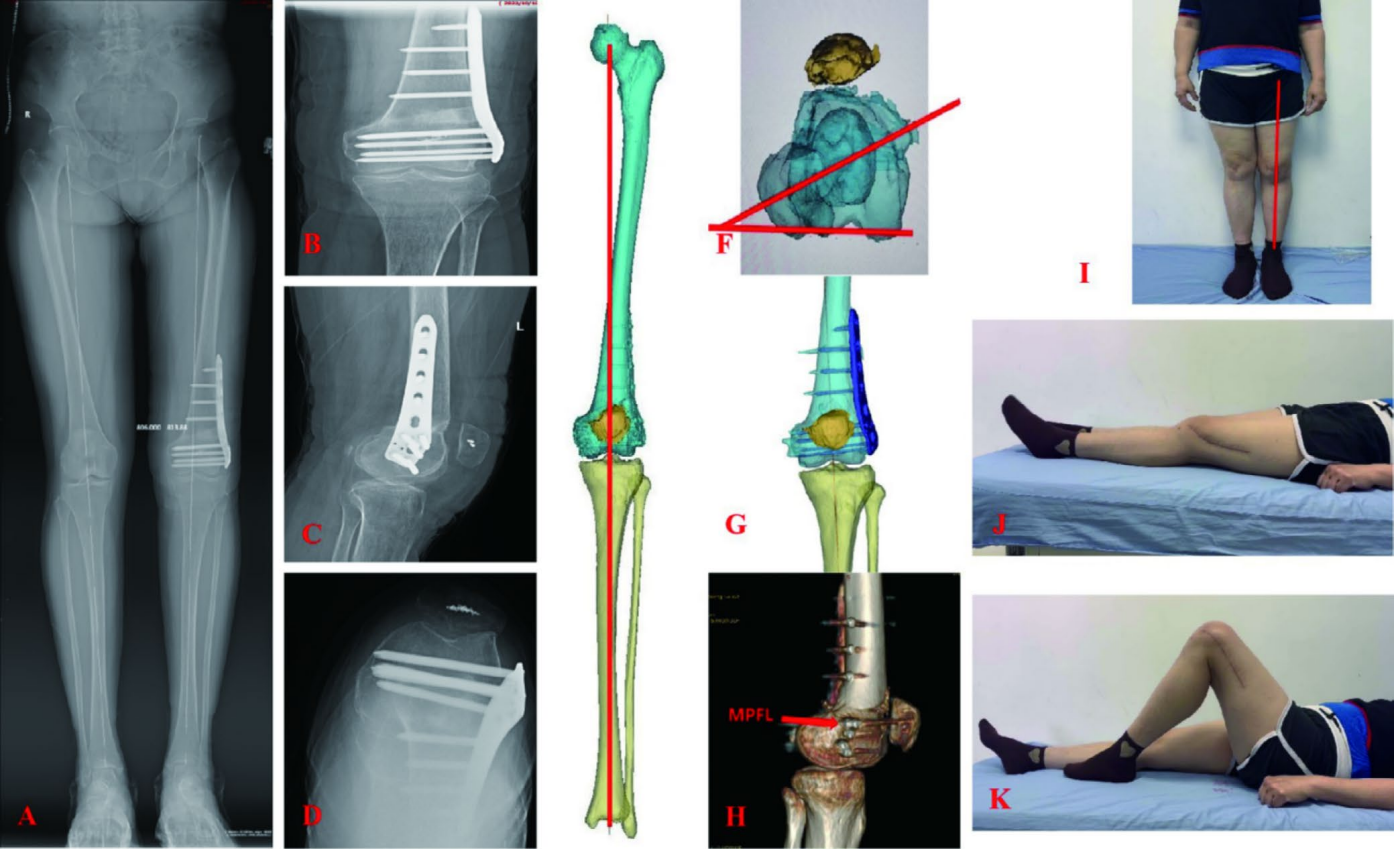
Postoperative photographs and fluoroscopy. (A) The postoperative standing X‐ray of the lower limbs. (B) The postoperative X‐ray of the knee in anteroposterior position. (C) The postoperative X‐ray of the knee in lateral position. (D) The postoperative X‐ray of the knee in 90° axial position. (E) The postoperative 3D‐CT showed that the lower limb alignment returned to normal. (F) The postoperative 3D‐CT showed that the femoral anteversion angle was 15°. (G) The postoperative 3D‐CT showed that the patellofemoral relationship returned to normal. (H) The postoperative 3D‐CT indicated that the femoral stop position of reconstructed MPFL was satisfactory. (I) Good function while standing. (J) Good function while knee extension. (K) Good function while knee flexion.

Postoperative DR Films and CT showed that the patellofemoral relationship and lower limb alignment returned to normal. There were no complications such as wound infection, nerve injury, vascular injury, fracture nonunion, or patella dislocation during the follow‐up period. Osteotomy clinical healing was achieved at 3 months after the operation. At 6 months after the operation, the patient could walk freely, the ROM of the knee joint was 0°–130°, and there was no swelling or pain of the knee joint. The patellar extrapolate fear test was negative, and no patellar dislocation occurred again. The postoperative HKA was 180°, mLDFA was 90°, and FAA was 15°. The WOMAC index was 8 at 6 months after the operation. The patient was very satisfied with the status of her left knee after 6 months postoperation, so she did not come back again for follow‐up. See Figure 4 for details.

## 5 Discussion

Patellar instability can be caused by a variety of factors, including soft tissue factors and bone factors [12]. MPFL is considered an important structure to maintain the stability of the medial patella. MPFL can provide 50%–60% inward binding force at 30° knee flexion [13]. Recent studies have shown that MPFL reconstruction alone is not effective for patients with patellar instability combined with skeletal structural abnormalities [14, 15]. Patellar instability is usually caused by multiple causes at the same time. The surgical plan should be made according to the location and degree of deformity, as well as the symptoms and demands of patients. Knee valgus deformity and lower limb rotation deformity are two pathogenic factors that are often overlooked [16].

Valgus knee alignment is one of the known causes of patellar instability, recurrent patella dislocations, and damage to the MPFL, requiring surgical reconstruction [17]. Valgus correction by DFO is a feasible surgical method for patellar instability and genu valgus. It has been suggested that 3° valgus of the lower limb alignment can lead to a 2.5‐fold increase in the risk of knee joint degeneration and a 5.9‐fold increase in the risk of cartilage damage [18]. Valgus force line of the lower extremity will increase the Q Angle, leading to patellar lateral deviation and abnormal tracking [19]. DFO can make the tibial tuberosity shift relatively inward by correcting the force line, thereby reducing the *Q* angle and improving patellar tracking. Deng et al. [20] treated recurrent patellar dislocation with FAA enlargement and genu valgus and by using DDFO combined with medial closing wedge distal femoral osteotomy (MCWDFO) combined with MPFL reconstruction, and achieved satisfactory clinical and imaging results. Therefore, some studies have suggested that DFO should be considered as early as possible if valgus deformity is accompanied by patellar instability and cartilage damage [21, 22]. DFO is divided into two different methods: MCWDFO and LOWDFO. There were no significant differences in long‐term outcomes and complication rates between the two groups. A meta‐analysis also demonstrated good clinical outcomes in patients with knee valgus combined with recurrent patellar dislocation in either MCWDFO or LOWDFO [23]. In this case, the left lower limb was about 7 mm shorter than the right lower limb. To avoid further shortening of the affected limb after surgery, we chose LOWDFO.

Biomechanical studies have shown that MPFL reconstruction alone is insufficient to combat the lateral stress caused by the distal internal rotation (increased over 20°) of the femur [24]. Another biomechanical study also found that the increased FAA would cause excessive outward traction on the patella, while DDFO could reduce the excessive outward traction force and thus relieve the intrinsic tension of the MPFL graft [25]. Therefore, derotational osteotomy is a more reliable treatment for patients with torsion deformity due to our latest knowledge. In recent years, DDFO has been gradually applied in clinic and has achieved good clinical effect [26, 27].

There were few reports on the cases with both knee valgus deformity and lower limb rotation deformity. In this case, after comprehensive consideration, we decided to treat the patient with CDFO.

CDFO, as a novel osteotomy procedure, has several main characteristics. First, it combines the advantages of LOWDFO and DDFO through the same surgical incision, which can not only correct femur rotation deformity but also deal with knee valgus deformity. Second, CDFO uses the TomoFix plate system and does not require additional internal fixation. Third, CDFO consists of LOWDFO and DDFO, both of which are familiar surgical procedures for orthopedic surgeons. It may be easily mastered by surgeons who have already performed LOWDFO or DDFO.

Imhoff et al. [28] introduced a laboratory cadaveric study: During derotational osteotomy of the distal femur, the knee varus/valgus deformity was corrected by different direction osteotomy lines. The oblique Angle of the osteotomy line was calculated by a mathematical model, and the osteotomy guide was designed according to the angle. The distal femoral osteotomy was performed with the assistance of the 3D printed guide. However, osteotomy with this guide can only solve the varus/valgus deformity. For the correction of torsion deformity, intraoperative rotation with two additional fixator pins and measurement with a goniometer remains required according to the traditional method.

Our study introduces a novel technical advancement by integrating 3D‐printed osteotomy guides to achieve precise multi‐planar correction (valgus, rotation, and limb length) in a single procedure. The integrating 3D‐printed osteotomy guide is the focus of this study. The traditional osteotomy guide only serves the function of osteotomy guidance, but not orthopedic function. During the traditional operation, repeated fluoroscopy remains needed to determine the orthopedic angle. In addition, the previous evaluation of patients' deformity is usually two‐dimensional, which makes it difficult to achieve a three‐dimensional comprehensive evaluation [29]. The personalized osteotomy and orthopedic integrated guide designed in this research is a comprehensive surgical plan based on the three‐dimensional model and has orthopedic effects. The guide can be closely fitted to the anatomic markers of the femoral lateral condyle, and the osteotomy direction can be further verified by intraoperative fluoroscopy.

In addition, one of the keys to the success of MPFL reconstruction is to accurately locate the femoral tunnel [30]. Schöttle's method has been used to locate the femoral tunnel in the past, but repeated intraoperative fluoroscopy is required. In our research, the femoral tunnel position was designed in advance on the integrating 3D‐printed osteotomy guide. The Kirschner wire was inserted along this point, and the opposite side was the femoral tunnel position. There was no need for fluoroscopy during the operation, which greatly shortened the operation time. Finally, with computer assistance, customized surgical plans were provided for the clinic, including rotation angle, distraction distance, osteotomy direction and depth, osteotomy position, MPFL femoral tunnel positioning hole and screw size. This method can greatly shorten the learning curve of the operation, shorten the operation time, reduce intraoperative fluoroscopy, reduce the amount of blood loss and complications, and improve the clinical effect.

We believe that proper patient selection, detailed preoperative planning, experience of the surgical team, and reasonable rehabilitation is important reasons for the success of this case. The follow‐up time of this case is short, and the long‐term curative effect needs to be further observed. Despite the success of CDFO treatment, more research and time are needed in the future to evaluate its long‐term efficacy and shortcomings. Another limitation is that the CT data used in the preoperative design of this case is non‐weight‐bearing CT, and the measurement data may have certain errors. In future studies, weight‐bearing CT data should be used for preoperative design if conditions permit.

## 6 Conclusion

To our knowledge, this rare pattern of patellar instability has not been previously described. Careful analysis of anatomic abnormalities is of great clinical significance and can better guide clinical treatment. CDFO may be an acceptable treatment for patellar instability with genu valgus and increased femoral anteversion angle.

## Author Contributions

**Bin Zhao:** writing – original draft. **Zijian Lian:** writing – review and editing. **Xuan Jiang:** writing – original draft, data curation. **Songqing Ye:** methodology, visualization. **Haohao Bai:** investigation, data curation, methodology. **Wei Luo:** conceptualization, funding acquisition, supervision. **Xinlong Ma:** conceptualization, supervision.

## Ethics Statement

This study was approved by the Ethics Committee of the authors' hospital (2022‐141). The patient and her families were provided informed written consent.

## Conflicts of Interest

The authors declare no conflicts of interest.
